# Clinical and Imaging Markers of Prodromal Parkinson's Disease

**DOI:** 10.3389/fneur.2020.00395

**Published:** 2020-05-08

**Authors:** Eldbjørg Hustad, Jan O. Aasly

**Affiliations:** ^1^Department of Neurology, St. Olavs Hospital, Trondheim, Norway; ^2^Department of Neuromedicine and Movement Science (INB), Faculty of Medicine and Health Sciences, Norwegian University of Science and Technology (NTNU), Trondheim, Norway

**Keywords:** Parkinson's disease, prodromal markers, LRRK2, DAT-SPECT, olfaction

## Abstract

The diagnosis of Parkinson's disease (PD) relies on the clinical effects of dopamine deficiency, including bradykinesia, rigidity and tremor, usually manifesting asymmetrically. Misdiagnosis is common, due to overlap of symptoms with other neurodegenerative disorders such as multiple system atrophy and progressive supranuclear palsy, and only autopsy can definitively confirm the disease. Motor deficits generally appear when 50–60% of dopaminergic neurons in the substantia nigra are already lost, limiting the effectiveness of potential neuroprotective therapies. Today, we consider PD to be not just a movement disorder, but rather a complex syndrome non-motor symptoms (NMS) including disorders of sleep-wake cycle regulation, cognitive impairment, disorders of mood and affect, autonomic dysfunction, sensory symptoms and pain. Symptomatic *LRRK2* mutation carriers share non-motor features with individuals with sporadic PD, including hyposmia, constipation, impaired color discrimination, depression, and sleep disturbance. Following the assumption that the pre-symptomatic gene mutation carriers will eventually exhibit clinical symptoms, their neuroimaging results can be extended to the pre-symptomatic stage of PD. The long latent phase of PD, termed prodromal-PD, represents an opportunity for early recognition of incipient PD. Early recognition could allow initiation of possible neuroprotective therapies at a stage when therapies might be most effective. The number of markers with the sufficient level of evidence to be included in the MDS research criteria for prodromal PD have increased during the last 10 years. Here, we review the approach to prodromal PD, with an emphasis on clinical and imaging markers and report results from our neuroimaging study, a retrospective evaluation of a cohort of 39 participants who underwent DAT-SPECT scan as part of their follow up. The study was carried out to see if it was possible to detect subclinical signs in the preclinical (neurodegenerative processes have commenced, but there are no evident symptoms or signs) and prodromal (symptoms and signs are present, but are yet insufficient to define disease) stages of PD.

## Introduction

Parkinson's disease (PD) is clinically defined by the presence of cardinal motor symptoms, bradykinesia in combination with at least one of rest tremor or rigidity (1). The cardinal motor symptoms depend upon progressive degeneration of the dopamine-containing neurons in the substantia nigra pars compacta (SNpc) (2). The histopathological hallmark of PD is the presence of Lewy bodies (LBs), fibrillar aggregates in which α-synuclein is a major constituent (3). Pathological studies have shown a strong correlation between the extent of Lewy Body related cell loss in the Substantia Nigra (SN) and the severity of bradykinesia (4). Nigrostriatal dopaminergic damage can be monitored by functional neuroimaging techniques, such as positron emission tomography (PET) or single photon emission tomography (SPECT) (2).

During the last 25 years the clinical-pathological concept of PD has been challenged. Pathological studies estimate 40–60% loss of dopaminergic cells and reduction of synaptic function by up to 80% before the appearance of motor symptoms meeting current PD criteria appear (4). The Braak hypothesis posit the spread of Lewy pathology in a caudal to rostral pattern, suggesting early involvement of the peripheral autonomic nervous system (5).

According to current diagnostic criteria, PD is clinically diagnosed when disease progression is already advanced. This latent phase, which can vary from 5 to more than 20 years is called the prodromal phase of PD (6, 7). In this phase symptoms or signs of PD neurodegeneration are present, but a classic clinical diagnosis based on fully evolved motor parkinsonism is not yet possible (8). This phase represents an opportunity for earlier diagnosis, investigation of the pathophysiological cascade and when disease- modifying treatment become available, to possibly slow or prevent the onset of motor symptoms in PD (9–11). Patients in the prodromal phase constitute the ideal candidates to participate in trials of neuroprotective therapies because of their wide therapeutic window and lack of symptomatic therapies (6). Thus, identification of individuals in this phase is a clinical and research priority (6, 12). We are in need of biomarkers for the early diagnosis of PD. NIH Biomarkers Definitions Working Group defines a biomarker as “a characteristic that is objectively measured and evaluated as an indicator of normal biological processes, pathogenic processes or pharmacologic responses to a therapeutic intervention” (13).

Ten years ago there were six known prodromal markers of PD present, rapid eye movement (REM) sleep behavior disorder, olfactory loss, constipation, depression and anxiety, erectile dysfunction and somnolence, none of which had more than two studies documenting diagnostic value (14). Today, because of an extensive research into prodromal PD, the number of markers with the sufficient level of evidence to be included in the MDS research criteria for prodromal PD, have increased and includes as well orthostatic hypotension, urinary dysfunction, possible subthreshold parkinsonism (UPDRS >3 excluding action tremor) / abnormal quantitative motor testing and clearly abnormal dopaminergic PET/SPECT (8, 14).

## Clinical Markers

### Non-motor Markers (Table 1)

#### Olfactory Loss

Hyposmia is one of the most common and best-characterized non-motor features and is often one of the earliest prodromal features to emerge (15, 16). The association of hyposmia with PD is widely accepted. About 80% of PD patients have impaired olfaction, which is in line with Braak‘s hypothesis of Lewy pathology in the olfactory bulb (Braak stage 1) (17). Hyposmia can be objectively quantified with standard tests such as 12-item Brief Smell Identification Test (B-SIT) (4, 18). However the likelihood of developing PD is unclear. In a recent review and meta-analysis investigating the association between hyposmia and PD, hyposmia was associated with a 3.84-fold risk of developing PD (16). In the Honolulu-Asia Aging Study, olfactory dysfunction was associated with an increased risk of PD; however, the association was significant only for the first 4 years of follow-up because of the lack of systematic measurements of smell in epidemiological studies (19, 20). Other investigators noted that, among first-degree relatives of patients with PD, olfactory dysfunction significantly correlated with development of PD within the subsequent 2–5 years (19). Ponsen et al. (21) found in their prospective cohort study of 361 non-parkinsonian, non-demented first-degree relatives of PD patients, that a two-step approach of initial olfactory testing followed by dopamine transporter (DAT)- SPECT scanning in individuals with hyposmia strongly increases specificity while retaining the high sensitivity associated with olfactory testing alone.

**Table 1:** Clinical non-motor markers of prodromal Parkinson's disease.

**Marker**	**References**
Olfactory loss	(15–21)
Constipation	(8, 19, 22–27)
Rem sleep behavior disorder	(28–34)
Excessive daytime somnolence	(13, 36, 37)
Depression/anxiety	(38–45)
Global cognitive deficit	(46–49)
Orthostatic hypotension	(46, 50, 51)
Erectile dysfunction	(52–54)
Urinary dysfunction	(54, 55)

#### Constipation

Characterized by infrequent stools, difficult stool passage, or both, is one of the first, most common and disabling NMS to develop during the prodromal phase (22, 23). Recently constipation was included in both the research criteria for prodromal PD diagnostics as one of the risk factors for future development of PD (8, 22). Pathological alpha-synuclein inclusions can be detected in the entire gastrointestinal tract as early as 20 years before the diagnosis of PD, supporting the Braak proposed model for the pathophysiology of alpha-synuclein aggregates in PD (Braak stage 1) and making constipation one of the earliest recognizable prodromal features (22, 24). In a recent review and meta-analysis estimating the magnitude of association between premorbid constipation and later diagnosis of PD, constipation was associated with a 2.27-fold increased risk of developing PD, compared with someone without, and the increase in risk persists over a decade prior to diagnosis (25). Abbot et al. (26) found that after adjustment for confounders men with fewer than 1 bowel movement per day had a 2.7-fold higher odds of developing PD compared to men with more frequent bowel movements In the Honolulu-Asia Aging Study cohort, the mean interval from bowel-movement abnormality to PD symptoms was 10 years (and it was 12 years to PD diagnosis) (19, 26). Gao et al. (27) found in their Health Professionals Follow-up Study and Nurse Health Study that infrequent bowel movements were associated with a higher future risk of PD in the next 6 years.

#### REM-Sleep Behavior Disorder

REM sleep behavior disorder (RBD) is a parasomnia characterized by dream-enacting behavior typically involving vocalizations or movements of the upper extremities, related to unpleasant dreams and loss of normal REM-sleep muscle atonia (28–30). RBD can be classified into an idiopathic form (iRBD) and a marker of prodromal neurodegeneration or a secondary form which occurs in patients already diagnosed with PD (31). RBD was first described in 1986 by Schenck et al. (29). Patient-reported questionnaires have been developed for identification of individuals with RBD, but similarly to patients with hyposmia, patients with RBD are often not aware of their symptoms. Accurate collateral history from a bed partner is usually necessary to make the diagnosis. In questionable cases or for individuals without bed partners, polysomnography can be obtained (29). Cohort studies indicate that iRBD convert to PD and other synucleinopathies such as dementia with Lewy bodies and multiple systems atrophy (28, 32). Pheno-conversion risk between 2 and 5 years is about 15–35%, and the risk may increase to 41% to 90.9% if extending the follow-up period up to 12–25 years, thus making iRBD to date the most specific clinical prodromal marker of PD (29, 33). When examining prodromal criteria as well as the independence of prodromal markers to predict conversion to PD or dementia with Lewy bodies, Fereshtehnejad et al. (34) found that diagnostic accuracy of the MDS research criteria for prodromal PD was high in the RBD population.

#### Excessive Daytime Somnolence

Excessive daytime somnolence (EDS) consists in the inability to maintain wakefulness during the day, with sleep occurring unintentionally or at inappropriate times (13). EDS is a well-known feature of advanced PD with a prevalence of 30–40% (35). Two published population-based studies looked at EDS as a potential prodromal symptom in PD. The first was the Honululu-Asia Aging study which report a 2.8-fold increased relative risk of developing PD in the future in men who reported a subjective sense of daytime sleepiness (36). The second a population based study (220,000 participants) found that those who reported having daytime napping of ≥1 h had a 1.5-fold increase risk of developing PD (37). In a recent study Abbot et al. (36) stained for α-synuclein (Lewy pathology) in multiple brain regions in a sample of 211 men and found that EDS was more common in the presence vs. absence of Lewy pathology (*p* = 0.034) and the association became stronger 36.7% [11/30], *p* = 0.023 when LP reached the anterior cingulate gyrus, insula mesocortex, and midfrontal, midtemporal, and inferior parietal neocortex (Braak stage 5) and 3-fold increase [51.9% [14/27], *p* < 0.001] with further infiltration into the primary motor and sensory neocortices (Braak stage 6) (36).

#### Depression/Anxiety

Depression and anxiety are relatively common features of PD. Descriptive studies as early as 1913 noted a personality type, described as particularly industrious, devoted to hard work, inflexible, punctual, cautious, and moralist to be associated with PD (38). This anecdotal concept of premorbid -parkinsonian personality is supported by the Minnesota Multiphasic Personality Inventory (MMPI) long-term historical cohort study, suggesting that an anxious personality trait may predict an increased risk of PD developing many years later (39).

Clinically significant depressive disturbances are found to occur in 40–50% of patients with PD (40, 41). The onset of depressive syndromes and their natural history do not parallel the course of the motor symptoms (40, 42). A higher incidence of depression in patients who were later diagnosed with PD, supports the hypothesis of there being a biological risk factor for depression in these patients (43). Depression in PD has been related to multiple neurotransmitter dysfunctions, including dopamine (SNpc), serotonin (raphe nuclei), and noradrenaline (locus coeruleus). The involvement of both raphe nuclei and locus coeruleus at Braak stage 2, might indicate depression as a prodromal symptom of PD (44). The relationship between depression and subsequent PD appears to be strongest in the immediate “premotor” years before diagnosis of PD. Retrospective case-control analysis of a population-based study from Rotterdam suggests that both anxiety and depression become significantly more common in patients only about 1–2 years before PD diagnosis (45).

#### Global Cognitive Deficit

Cognitive deficits was associated with increased PD risk in two prospective studies investigating global cognition and cognitive decline (46). Darweesh et al. (47) found in a population-based cohort study including 7,386 participants of the Rotterdam Study with median 8.3 years of follow-up, poor baseline cognitive functioning indicated the probable onset of parkinsonism and probable Parkinson disease (47). Schrag et al. (48) analyzed data from 8,166 patients aged older than age 50 years with incident diagnosis of PD and 46,755 controls looking at likelihood ratios, sensitivity, specificity, and positive and negative predictive values for individual symptoms and combinations of presentations including cognitive decline. They found that cognitive decline was significantly associated with PD within 5 years before diagnosis. Based on those two studies and the study from Weintraub et al. (49) who found that global cognition was numerically, but not statistically worse in individuals with hyposmia and incident PD compared with those who remained PD free, global cognitive deficit was recently added as a prodromal marker in the MDS research criteria for prodromal PD (46).

#### Orthostatic Hypotension

Neurogenic orthostatic hypotension (nOH), the hallmark feature of degeneration of the autonomic nervous system, refers to clinically diagnosed orthostatic hypotension (OH) with confirmation based on quantitative assessments of supine/sitting and standing blood pressure drop with alternative causes of OH (dehydration, cardiac disease, autonomic neuropathy, medication, etc.) eliminated after comprehensive clinical assessment (46, 50). Symptomatic OH is based on clinical OH diagnosis or a positive orthostatic hypotension questionnaire without comprehensive diagnostic investigation regarding the cause (46). The work by the US Autonomic Disorders Consortium showed that nOH in the presence of rapid eye movement sleep behavior disorder and reduced olfaction carries a 10 percent annual cumulative risk of developing Parkinson disease and dementia with Lewy bodies (51). The MDS Research Criteria for Prodromal PD consider nOH as one of the key features of prodromal PD (8). Recently new levels of diagnostic certainty for neurogenic and symptomatic orthostatic hypotension have been added to the criteria (46).

#### Erectile Dysfunction

Dysautonomia is common among all synucleinopathies and limited dysautonomia may predate the motor symptoms by up to 20 years (52). Although erectile dysfunction (ED) is an autonomic symptom only a few studies have documented the frequencies of ED in PD. In a large-scale cohort with 32,616 US men, Gao et al. (53) observed that erectile dysfunction was prevalent among PD patients and that ED antedates PD diagnosis by many years. Postuma et al. (54) reported from a prospective follow up in a RBD cohort that ED was significantly abnormal up to 5 years before the development of a defined neurodegenerative disease. However, Hasan et al. (52) did not find ED to be a premotor symptom among PD cases.

#### Urinary Dysfunction

Schrag et al. (55) found in a case control study a relative risk of 1.9 for urinary dysfunction at 5 years before PD diagnosis compared with controls (*n* = 25 544). Among patients with idiopathic RBD, symptoms of urinary frequency were documented up to 7 years before conversion to PD, with an extrapolated prodromal interval of 13 years (54). The specificity of this marker is, however, relatively low.

### Motor Markers

The UPDRS was developed as a rating scale within PD (56). According to MDS prodromal PD criteria, possible subthreshold parkinsonism on expert examination defined as a UPDRS score >3 excluding action tremor or MDS-UPDRS score >6, excluding postural and action tremor, is a clinical motor marker for prodromal PD (8).

UPDRS first becomes abnormal 4.5 years before diagnosis. Voice and face akinesia seem to be the first signs to develop, followed by rigidity, gait abnormalities, limb bradykinesia and finally tremor (68). Simple quantitative motor tests, may be able to identify parkinsonism earlier than subjective examination (68). Wearable or smartphone-based sensor technologies have been considered for continuous monitoring. However, sensor-based quantitative motor and non-motor markers such as cardiac and/ or autonomous dysfunction in prodromal PD require further prospective evidence and standardization of methods (46).

### Fluid, Tissue and Genetic Markers (Table 2)

Altered α-synuclein metabolism in the central nervous system has a central role in the pathogenesis of PD and several studies have focused on determining α-synuclein species in different fluids and tissues. The α-synuclein is mainly expressed by neuronal cells as a cytoplasmic protein in its native form or in the oligomeric, phosphorylated form. However, because of its access to the extracellular space, it can be detected in cerebrospinal fluid (CSF) (13). Longitudinal changes in CSF α-synuclein and other biomarkers in PD have been examined in different cohorts with different results (69–72). In a recent study by Mollenhauser et al. (61) CSF-α-synuclein in drug-naïve PD, healthy controls, and prodromal PD in the Parkinson's Progression Markers Initiative (PPMI) up to 36-month follow-up was analyzed. According to the results, CSF α-synuclein decreases early in the disease, preceding motor PD. However, CSF- α-synuclein does not correlate with progression and therefore does not reflect ongoing dopaminergic neurodegeneration. Blood has been a disappointing target to-date because red cells contain large quantities of α-synuclein, obscuring any theoretical difference in levels between patients and controls (73).

**Table 2:** Fluid, tissue, and genetic markers of prodromal Parkinson's disease.

**Method**	**Biomarker**	**Value**	**References**
**Fluid**			
Blood			
	*Urat*	Low plasma urate levels in men are associated with higher PD risk	(46, 57–59)
	*NfL*	Biomarker panel in combination with CSF α-synuclein species	(60)
CSF			
	*α-synuclein*	No correlate with PD progression	(61)
	*NfL*	Biomarker panel in combination with CSF α-synuclein species	(60)
**Tissue**			
Skin			
	*α-synuclein*	Phosphorylated α-synuclein in skin biopsy are sensitive (55–100%) and highly specific (>90%) for PD and prodromal PD (idiopathic RBD)	(46, 62–64)
Submandibular gland			
	*α-synuclein*	Sensitivity of the marker depends on the number and location of tissue samples and specificity may vary between biopsy techniques	(65, 66)
**Genetic**			
	*G2019S LRRK2 mutation*	Mutation carriers without motor symptoms of PD represents a unique opportunity for studying the prodromal stage of PD	(67)

In large prospective studies, low plasma urate levels in men have repeatedly been shown to be associated with higher PD risk and are recently proved sufficiently sensitive and specific to be included as a risk marker in the MDS Research Criteria for Prodromal PD (46, 57–59).

In recent years there has been an increasing interest in Neurofilament light chain (NfL) as a biofluid biomarker for PD. Oosterweld et al. (60) found that CSF and serum NfL levels in combination with CSF α-synuclein species(phosphorylated-/total α-synuclein, and oligomeric-/total α-synuclein) may serve as a biomarker panel for discrimination of PD patients compared with controls.

The GI tract harbors the largest nervous system outside the CNS accessible for biopsy-taking by endoscopy. However, recent studies show conflicting results regarding α-synuclein detection in GI tract as a potential biomarker of PD. Schneider et al. (74) conclude in their review that data retrieved so far on alpha synuclein aggregations in the GI tract/salivary glands are still unsatisfactory in terms of specificity and sensitivity and are therefore not suitable to serve as a robust diagnostic biomarker (75).

Phosphorylated α-synuclein in skin biopsy has been shown to be sensitive (55–100%) as well as highly specific (>90%) for PD and prodromal PD (idiopathic RBD). Similarly, biopsy of the submandibular gland shows considerable promise. However, sensitivity of this marker depends on the number and location of tissue samples and the specificity may vary between biopsy techniques. Prospective studies proving predictive value are still lacking (46, 62–66).

Although there are promising approaches in fluid and tissue biomarker research, no biofluid or histological marker has proven sufficiently sensitive and specific to be included as a prodromal marker in the MDS research criteria for prodromal PD. Currently there are no validated biomarkers to assist in diagnosing PD or determining its neuropathological progression (76). So far, the prodromal criteria are composed of clinical and imaging signs (77).

#### Genetic Cohorts

Evidence from family and twin studies in addition to advances in molecular genetics have indicated important genetic contributions to the pathogenesis of PD (78). Although, monogenic causes of PD, such as autosomal dominant mutations in the *SNCA, LRRK2*, or *VPS35* genes, is limited to small minority of individuals, asymptomatic carriers of mutations that cause monogenic forms of PD provide the clearest information on the development of prodromal features (10, 17, 79).

The most common monogenic cause of PD is mutation of the autosomal dominant Leucine Rich Repeat Kinase (*LRRK2*) gene, a complex gene whose role in neurodegeneration is not completely understood. The G2019S mutation in the *LRRK2* gene, represents the most common pathogenic mutation identified in PD worldwide, accounting for up to 1–6% of sporadic and 3–19% of familial PD with even higher frequencies in Ashkenazi Jews (11, 80). At present there are no sensitive methods to identify those likely to develop the disease. Non-manifesting carriers (NMC) are considered to have an increased risk, G2019S penetrance range between 30 and 80% at age 80, for future development of the disease (11). Phenoconversion from a motorically asymptomatic to an affected state probably reflects an age-associated failure to compensate for kinase dysfunction (81). Once manifest, the motor features of *LRRK2*- PD are largely indistinguishable from idiopathic PD (10). The identification and follow-up of carriers of the *LRRK2*- G2019S mutation who still have not developed motor symptoms of PD represents a unique opportunity for studying the prodromal stage of PD (67). Mirelman et al. (82) has been the first to evaluate the MDS Research Criteria for Prodromal PD in carriers of the *LRRK2*- G2019S mutation and the first among Ashkenazi Jews. According to their results, the criteria had high sensitivity and specificity in identifying prodromal PD in this high- risk unique cohort.

Glucocerebrosidase (*GBA*) mutations are together with *LRRK2* variants, the most common genetic risk factors for late-onset PD. About 5–10% of PD patients have mutations in the *GBA1* gene and *GBA* mutation raises as high as nearly 7-fold of odds ratio for PD in its carriers.

## Imaging Markers

Neuroimaging of genetic PD can provide unique opportunities to investigate changes occurring in the pre-symptomatic period in asymptomatic carriers (83). Although dopamine levels cannot be measured directly by using imaging, various methods can be used to assess altered function of nigrostriatal dopaminergic neurons terminals. The most easily accessible approach is the use of markers for the dopamine transporter (DAT). Functional cerebrum imaging using tracers, that can penetrate the blood-brain barrier, can identify diseased areas in the cerebrum with either positron emission tomography (PET) or single photon emission computed tomography (SPECT) (84).

Dopamine transporter single photon emission tomography (DAT-SPECT) is a neuroimaging method providing a semiquantitative assessment of striatal dopaminergic deafferentation and is a well-established method for the assessment and investigation of PD (11, 85). In patients with PD, DAT-SPECT shows decreased striatal DAT uptake, indicating substantia nigra dopaminergic dysfunction that is more marked in the putamen than in the caudate nucleus (12). Studies in unaffected subjects with PD mutations, hyposmia, and a first-degree relative with PD or RBD demonstrate abnormal dopaminergic imaging in advance of motor symptoms (86).

Cohort studies comparing RBD subjects to healthy controls have demonstrated that around 20–40% of RBD patients have abnormal DAT imaging (17). In one of the largest studies using DAT- SPECT, a prospective study of 43 iRBD patients, Iranzo et al. (12) found that decreased striatal DAT uptake (^123^I-FP-CIT binding) and substantia nigra hyperechogenicity might be useful markers to identify individuals at increased risk for developing synucleinopathies. After a follow up of 2.5 years, there was a pathologically reduced ^123^I-FP-CIT binding in 17 (40%) of 43 participants and substantia nigra hyperechogenicity in 14 (36%) of 39 participants. A total of 63% of the participants had reduced ^123^I-FP-CIT binding or substantia nigra hyperechogenicity at baseline. Of these, 30% developed a neurodegenerative disorder (five PD, two dementia with Lewy bodies, and one multiple system atrophy). When examining iRBD patients with serial ^123^I-FP-CIT SPECT Iranzo et al. (87) found a decline in striatal tracer uptake reflecting a progressive nigrostriatal dopaminergic dysfunction. The DAT deficit seen in RBD is less severe than in established PD suggesting that dopaminergic imaging may have the potential to quantify progression through the prodromal phase (17, 88, 89). In a recent study by Bae et al. (90) they found that 3.0-T susceptibility-weighted MR imaging showed alterations of nigral hyperintensity in patients with iRBD that corresponded to DAT SPECT findings. However, future studies with a larger number of study subjects are recommended since 36.1% of the patients with iRBD showed discordance between the findings.

The first study performing DAT-SPECT in a cohort of unaffected carriers of the G2019S mutation was made by Sierra et al. (91) were they report abnormal DAT imaging in 43.7% of the participants.

Sossi et al. (92) examined changes in dopamine turnover in the asymptomatic PD phase using PET imaging with 18F-fluorodopa and found dopamine turnover to be elevated in asymptomatic mutation carriers at increased risk of PD. Wile et al. (93) did two cross-sectional PET studies showing that LRRK2 mutation carriers without manifest Parkinson's disease had greater 18F-fluorodopa uptake and dopamine transporter binding than did individuals with sporadic Parkinson's disease increased serotonin transporter binding in the striatum, brainstem, and hypothalamus, possibly reflecting compensatory changes in serotonergic innervation preceding the motor onset of Parkinson's disease. In another study Liu et al. (94) used the PET tracer N-^123^C-methyl-piperidin-4-yl propionate to scan for acetylcholinesterase activity in 4 patients with LRRK2 Parkinson's disease, 16 LRRK2 mutation carriers without Parkinson's disease, eight patients with idiopathic Parkinson's disease, and 11 healthy controls. They found that LRRK2 mutations are associated with significantly increased cholinergic activity in the brain in mutation carriers without Parkinson's disease compared with healthy controls.

In the clinical setting, DAT- PET has several advantages over DAT- SPECT like superior DAT selectivity, shorter static imaging protocols without the need for pharmacological thyroid protection, better image resolution with PET, and possibility to obtain quantitative outcome measures with full dynamic PET acquisitions, when required (95).

According to a update of the MDS Research Criteria for Prodromal Parkinson's Disease by Heinzel et al. (46) several imaging approaches have potential as sensitive and specific markers of prodromal PD as suggested by associations with RBD, GBA, or LRRK2 mutation carriers, Dementia with Lewy Bodies, and PD. These promising neuroimaging techniques include 11C-donepezil PET/CT (cholinergic (parasympathetic) gut innervation), 123I-metaiodobenzylguanidine scintigraphy (cardiac sympathetic denervation), susceptibility-weighted and neuromelanin-sensitive MRI (dorsal nigral hyperintensity; integrity of pigmented neurons of the locus coeruleus), coeruleus), 11Cmethylreboxetine PET (noradrenergic nerve terminals originating in the locus coeruleus), structural connectivity and functional MRI (striatal or whole-brain function) (17, 46, 96–98).

In a recent study by our group, a retrospective evaluation of a cohort of 39 participants who underwent DAT-SPECT scan as part of their follow up by movement disorder expert (JOA) at the department of Neurology at St. Olav‘s Hospital in Trondheim were performed. The report was given prior to the imaging studies. The material has been described in previous reports (80, 99–101). Our objective was to assess whether a combination of systematic clinical testing and different imaging techniques in familial PD cases could detect subclinical signs in the preclinical and prodromal stages of PD. We characterized the cohort of 39 participants with visual analysis of DAT- SPECT imaging to assess patterns of dopaminergic degeneration. Participants were divided into five groups based on the Movement Disorders Society (MDS) Research Criteria for Prodromal PD (8, 73). (1) healthy, (2) preclinical *LRRK2* PD (*LRRK2*- mutation- carriers without clinical symptoms), (3) prodromal *LRRK2* PD (*LRRK2* mutation carriers with presence of early symptoms and signs before PD diagnosis is possible), (4) clinical *LRRK2* PD (*LRRK 2* carriers with diagnosis of PD based on the presence of classical motor signs) and (5) clinical PD (idiopathic PD).

Clinical assessment included the Unified Parkinson's Disease Rating Scale (UPDRS) part III (motor part). Participants without a PD diagnosis, were divided into preclinical *LRRK2* PD (UPDRS III <5) or prodromal *LRRK2* PD (UPDRS III 5–10) based on the UPDRS part III score, as seen above.

The participants were distributed as follows: 1 healthy, 18 preclinical *LRRK2* PD,12 prodromal *LRRK2* PD, 5 clinical *LRRK2* –PD, and 3 clinical idiopathic PD (iPD). We assume that pre-symptomatic gene mutation carriers will eventually exhibit clinical symptoms and, thus, the imaging results can be extended to the pre-symptomatic stage of PD (92).

DAT-SPECT scans were visually categorized by 1 observer according to predefined visual patterns of dopaminergic degeneration (102). It has been suggested that diagnostic accuracy in DAT-SPECT scans might be highly dependent on the reviewers experience as currently interpretation is mainly visual and therefore semi- quantitively and subjective (11). To avoid bias the observer was blinded to diagnosis and clinical features. The results were graded as normal (grade 1) or abnormal (grade 2–5), distinguishing an almost normal, symmetrical tracer uptake with a discrete reduction in one or both putamina (grade 2“eagle wing”), an asymmetric tracer uptake with normal or almost normal uptake in the putamen of one hemisphere and reduced uptake in the contralateral putamen (grade 3“mixed type”), a posterior-anterior degeneration pattern (grade 4 “egg shape”) and severe degeneration pattern (grade 5 “burst striatum”).

A correlation of the scan findings with the clinical symptoms and diagnosis was performed. Interobserver disagreement to the scan findings was considered.

In the preclinical *LRRK2* PD group, 28 percent (5/18) of the participants have normal DAT-SPECT scans and 72 percent (13/18) have abnormal DAT-SPECT scans grade 2 with an almost normal, symmetrical tracer uptake with a discrete reduction in one or both putamina.

In the prodromal *LRRK2* PD group, all of the participants have abnormal DAT-SPECT scans: 31 percent (4/13) grade 2, 38 percent (5/13) grade 3 with an asymmetric tracer uptake with normal or almost normal uptake in the putamen of one hemisphere and reduced uptake in the contralateral putamen and 31 percent (4/13) grade 4 with a posterior-anterior degeneration pattern.

**Figure 1:**
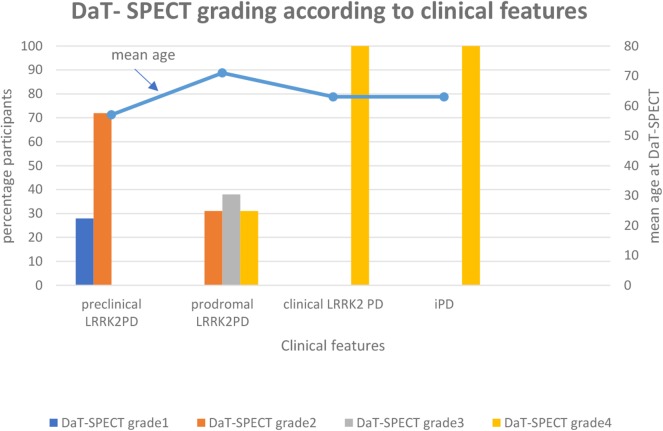
In the preclinical *LRRK2* PD group (UPDRSIII <5), 28 percent (5/18) of the participants have normal DAT-SPECT scans and 72 percent (13/18) have abnormal DAT-SPECT scans grade 2 with an almost normal, symmetrical tracer uptake with a discrete reduction in one or both putamina. In the prodromal *LRRK2* PD group (UPDRS III 5–10), all of the participants have abnormal DAT-SPECT scans: 31 percent (4/13) grade 2, 38 percent (5/13) grade 3 with an asymmetric tracer uptake with normal or almost normal uptake in the putamen of one hemisphere and reduced uptake in the contralateral putamen and 31 percent (4/13) grade 4 with a posterior- anterior degeneration pattern. In the clinical *LRRK2* group and idiopathic PD group all of the participants have abnormal DAT-SPECT scans grade 4.

In the clinical *LRRK2* PD group and idiopathic PD group, all participants have abnormal DAT -SPECT scans grade 4 (Figure 1).

Among the participants with normal DAT-SPECT scans, 100 percent (5/5) have preclinical *LRRK2* PD.

Among the participants with light abnormal “eagle wing” DAT -SPECT scans, 72 percent (13/18) have preclinical *LRRK2* PD, 22 percent (4/18) have prodromal *LRRK2* PD and the one control patient accounted for 6%.

Among the participants with moderate abnormal “mixed type” DAT-SPECT scans, 100% (5/5) have prodromal *LRRK2* PD.

**Figure 2:**
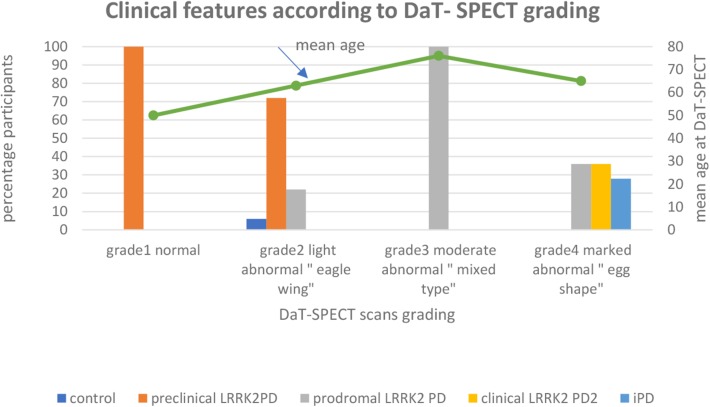
Among the participants with normal DAT-SPECT scans, 100 percent (5/5) have preclinical *LRRK2* PD. Among the perticipants with light abnormal “eagle wing” DAT-SPECT scans, 72 percent (13/18) have preclinical *LRRK2* PD, 22 percent (4/18) have prodromal *LRRK2* PD and the one control patient accounted for 6%. Among the participants with moderate abnormal “mixed type” DAT-SPECT scans, 100% (5/5) have prodromal LRRK2 PD. Among the participants with marked abnormal “egg shape” DAT-SPECT scans, 36 percent (4/11) have prodromal *LRRK2* PD, 36 percent (4/11) have clinical *LRRK2* PD and 28 percent (3/11) have idiopathic PD.

Among the participants with marked abnormal “egg shape” DAT-SPECT scans, 36 percent (4/11) have prodromal *LRRK2* PD, 36 percent (4/11) have clinical *LRRK2* PD and 28 percent (3/11) have idiopathic PD (Figure 2).

## Conclusion

New research criteria for prodromal PD are a promising tool to identify cases of incident PD over 5 years, arguing for their usefulness in defining target populations for disease-prevention trials (103). There are a wide variety of proven markers of prodromal PD with different predictive abilities and different lead times. The field of prodromal PD is rapidly expanding, with new diagnostic markers discovered each year (104). It is now possible to define with reasonable certainty the probability that a specific person has prodromal PD (104). According to the MDS research criteria for prodromal PD published in 2015, a Bayesian naive classifier approach is used to estimate the likelihood that an individual has prodromal PD by considering age and predictive information from risk and prodromal markers (8, 46). Once neuroprotective therapy has been developed, systematic screening for prodromal PD and resultant prompt treatment could even prevent clinical PD from ever becoming clinically relevant (104). However, clinical PD is a heterogeneous and complex disease with many different possible etiologies. Some evidence suggests that the presence of RBD, symptomatic hypotension, and cognitive deficits is associated with a more malignant PD phenotype, with a different prodromal state. Similarly, patients with *LRRK2* mutations often have prominent prodromal gait deficits, and *LRRK2* carriers with synuclein pathology exhibit more cognitive impairment, anxiety, and orthostatic hypotension than those without which will likely have a different prodromal state. Heterogeneity of prodromal states should be further investigated and may be important for targeted trial recruitment (46). Age and sex may impact the diagnostic accuracy of prodromal PD as well as the predictive properties of single risk and prodromal markers of PD and was taken into account when the MDS criteria of prodromal PD to improve the accuracy of PD prediction was revised (46, 105). Some PD patients suffer more from non-motor symptoms (106). Data quality of prodromal markers and their sensitivity and specificity may depend on assessment methods used. Not least, the accuracy of the PD diagnosis may vary between studies such as in register studies using medical record data (46, 107). Pillotto et al. (108) evaluated the MDS prodromal PD criteria in two independent prospective studies. They found that the criteria have low sensitivity and positive predictive values, but high specificity and negative predictive values in their cohorts. It is therefore required thorough quantitative/objective and specific diagnostic testing to yield the diagnostic accuracy necessary for selecting populations at risk for the first intervention trials in prodromal PD. Further research and refinements are needed for optimizing cut-offs and establishing appropriate means to account for the age-related normal changes, missing data, or incomplete assessment the diagnostic accuracy necessary for selecting populations at risk for the first intervention trials in prodromal PD.

## Author Contributions

EH designed and conceptualized the study, analyzed the data, and drafted the manuscript for intellectual content. JA designed and conceptualized the study, collected the data, analyzed the data, and revised the manuscript for intellectual content.

## Conflict of Interest

The authors declare that the research was conducted in the absence of any commercial or financial relationships that could be construed as a potential conflict of interest.
